# Musings on Sketches, Artists, and Mosquito Nets

**DOI:** 10.3201/eid2008.AC2008

**Published:** 2014-08

**Authors:** Byron Breedlove

**Affiliations:** Centers for Disease Control and Prevention, Atlanta, Georgia, USA

**Keywords:** art science connection, emerging infectious diseases, art and medicine, James Abbott McNeill Whistler, Man at Table beneath Mosquito Net, Musings on sketches, artists, and mosquito nets, mosquitoes, mosquito nets, West Point, Aesthetic Movement, vector-borne disease, malaria, about the cover

**Figure:**
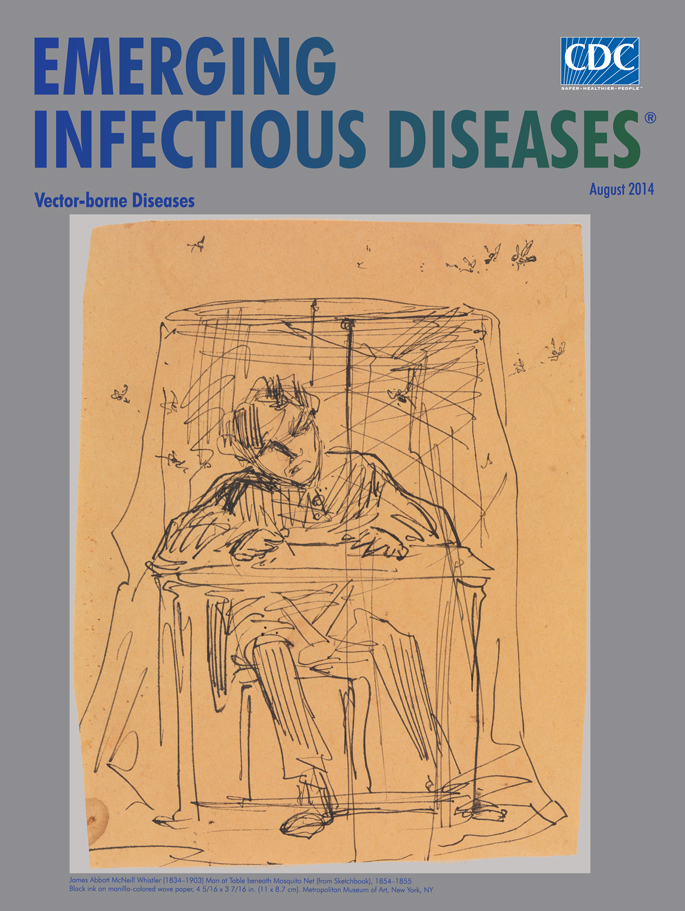
**James Abbott McNeill Whistler (1834–1903) Man at Table beneath Mosquito Net (from Sketchbook), 1854–55. Black ink on manila-colored wove paper, (4 5/16 × 3 7/16 in/11 × 8.7 cm)** Gift of Margaret C. Buell, Helen L. King, and Sybil A. Walk, 1970 (1970.121.40). Image copyright © The Metropolitan Museum of Art. Image

James Abbott McNeill Whistler was born in Lowell, Massachusetts, on July 11, 1834. When he was 9 years of age, his family moved to St. Petersburg, Russia, and there he studied drawing at the Imperial Academy of Science.

He later attended the United States Military Academy at West Point for 3 years, where he excelled in drawing classes. Because Whistler proved more adept at accumulating 218 demerits than at completing course work or complying with the disciplinary code, West Point Superintendent Colonel Robert E. Lee was obliged to dismiss the young cadet from the academy in 1854.

After a short unhappy stint as a draftsman, Whistler briefly lived with the Winan family in Baltimore, Maryland, where, he “would also ruin all our best pencils, sketching not only on the paper, but also on the smoothly finished wooden backs of the drawing-boards. . . .” During this time, he began experimenting with techniques, themes, and subjects. Art researcher Nancy Dorfman Pressly wrote that “Drawing less from his imagination, he began to look more carefully around him, catching a moment of unguarded behavior or simply the amusing attitudes of people in everyday situations.”

In 1855, Whistler moved back to Europe where he began to establish himself as a painter in Paris and London. He completed more than 500 paintings not only in oils, but also in pastels and watercolors. Among the best known of his paintings are *Nocturne: Blue and Gold – Old Battersea Bridge* and *Arrangement in Grey and Black No.1*, commonly and mistakenly called *Whistler’s Mother*.

In addition to capturing everyday situations, Whistler became recognized for his portrait paintings, including one of Thomas Carlyle. He was celebrated for his etchings of family members, mistresses, and street scenes, and some art historians suggest that the quality of his work matched that from the deft hand of Rembrandt.

Whistler was a leader in the Aesthetic Movement, writing and lecturing on the philosophy of “art for art’s sake.” His work later provided the inspiration for Oscar Wilde's novel *The Picture of Dorian Gray* (1891). Whistler died on July 17, 1903.

During the last decades of the artist’s life, a number of great advances in understanding disease transmission occurred. In 1880, Charles Louis Alphonse Laveran, a French physician, identified the protozoan parasite that causes malaria. Seven years later, Ronald Ross and his team discovered the malaria protozoa in anopheles mosquitoes.

Also around this time, in 1900, Walter Reed and a team of colleagues demonstrated that yellow fever was transmitted by mosquitoes. On New Year’s Eve, in a letter to his wife, Reed wrote “[I]t has been permitted to me and my assistants to lift the impenetrable veil that has surrounded the causation of this most dreadful pest of humanity and to put it on a rational and scientific basis.”

In *Man at Table beneath Mosquito Net*, Whistler himself might be the subject of this black ink drawing, part of a collection of such drawings from 1854–55. Whistler captures the continued struggle of humans versus biting and stinging insects, including those that transmit vector-borne pathogens, from an intimate perspective.

Despite the mosquitoes teeming around him, the man is able to sketch intently and without worry, sheltered by the confines of his personal impenetrable veil. The flurry of cross-hatched, finely scrawled lines in these ephemera could be seen to mimic a mosquito’s flight path but this was simply a common technique that Whistler used in his sketches.

Mosquito nets, particularly bed nets or sleeping nets, have, in some shape and form, been used for thousands of years. Herodotus described how people living in marshes in ancient Egypt fished with nets during the day then slept under the same nets to repel insects. Today, pyrethroid-treated mosquito nets are used extensively in malaria-endemic countries in Africa, yielding life-saving returns for little cost.

Personal measures such as using insect repellent, covering exposed skin with clothing, and using mosquito nets also provide simple, cost-effective—albeit not foolproof— protection. Various antimalarial drugs help combat this problem, but none is 100% protective, and they may cause adverse effects. Neither method is perfect but both are essential tools for preventing malaria.

The World Health Organization reported that in 2012, 207 million cases of malaria occurred, causing an estimated 627,000 deaths, mostly in children under 5 years of age. Today, another aspiring young artist working under his or her mosquito net may be sketching formative works that will someday inspire conversation and comment, and be a prelude of greater things to come, as did Whistler’s *Man at Table beneath Mosquito Net.*
